# Monitoring refugee health: Integrative approaches using surveys and routine data

**DOI:** 10.25646/7861

**Published:** 2021-03-31

**Authors:** Kayvan Bozorgmehr, Claudia Hövener

**Affiliations:** 1 Department of Population Medicine and Health Services Research, School of Public Health, Bielefeld University, Bielefeld, Germany; 2 Section for Health Equity Studies and Migration, Department of General Practice and Health Services Research, University Hospital Heidelberg, Heidelberg, Germany; 3 Robert Koch Institute, Berlin Department of Epidemiology and Health Monitoring

The Federal Health Reporting of the future will face the challenge of considering not only social developments such as demographic ageing but also the increasing diversity of society, for example with respect to migration. Since the first focus report on ‘Migration and Health’ has been published by the Federal Health Reporting in 2008 [1], major advances have been made in the underlying data sources. Health monitoring at the Robert Koch Institute, amongst other efforts, has been further advanced to increase its sensitivity to migration [2]. However, the inclusion of certain migrant groups has remained a challenge. Representative health studies currently do not systematically take into account migrant workers in precarious employment, people without an official residence permit and refugees. Particularly in the case of refugees, there are obvious structural weaknesses that have resulted in an incomplete information base. Germany has been a destination for people seeking international protection to varying degrees since the 1990s. Despite this, nationwide data that are comparable over time and space on the health and care of this migrant population are virtually non-existent.

There are many reasons for this gap. During the asylum procedure, refugees are initially accommodated in central, state-run reception facilities before being transferred to collective accommodation run by each district. During this process, they are not registered in official population registries and are hence practically not accessible by conventional sampling approaches. This group of people is extremely diverse with respect to their country of origin, languages spoken, reasons for fleeing and route taken, as well as residency prospects and socioeconomic background. It is therefore impossible to conduct surveys without a linguistic, cultural and contextual adaptation of survey instruments. In addition, due to the high level of migration dynamics and spatial displacement, there is no overview of the entire refugee population (denominator population), which is an essential parameter for health monitoring among refugees [3]. There are also substantial limitations when using routine healthcare data. During their initial stay at central, state-run reception facilities, refugees usually receive primary medical care in the facilities’ own outpatient clinics. However, routine data is not collected and collated uniformly in these. Those who use regular, external healthcare services, while staying at a reception facility or after transfer to the districts, are only identifiable as refugees in health insurance provider data in regions which provide refugees with an electronic health card (eGK). At the same time, refugees are generally only issued an eGK after leaving the reception facilities. This can be up to 18 months after their arrival in Germany or when the entire asylum application process has been completed.

Due to these factors, data on refugee health and provision of care remains incomplete and is based almost exclusively on local individual studies and surveys of limited duration that are generally incompatible with the principles of health reporting. An important exception is the survey of refugees established in 2016 by the Institute for Employment Research (IAB), the Federal Office for Migration and Refugees (BAMF) and the Socio-Economic Panel (SOEP), which uses a sample from the Central Register of Foreigners to supplement the established SOEP surveys. While this data source provides information on the living situation of refugees, the number of indicators on health and healthcare provision is limited.

The situation is similar in many other European countries. A review analysing the integration of migrants into health information systems and the availability of corresponding data in the European Region of the World Health Organization (WHO) revealed that only 23 of the 53 WHO member states have systematic and routine approaches to collecting health data on migrants [4]. Countries with nation-wide standardised registers were able to examine key aspects of health such as mortality, life expectancy and morbidity as well as collect data relating to pregnancy and childbirth for refugees, and compare these to other groups such as people with a migrant background or those without a migrant background. Nevertheless, there was a lack of feasible approaches to a systematic inclusion of migrants and particularly refugees in existing health surveys that would allow the collection of self-reported and more complex aspects of health. In many cases, systems for recording notifiable infectious diseases were the only sources of routinely available data with which to assess the health of refugees. Routine medical data from individual clinics were also frequently used, yet such data allowed only limited comparisons with other settings.

How can we ultimately improve the availability of information on the health of refugees as well as the integration of this information into health reporting at municipal, state and federal levels? This question, which is highly relevant internationally, is addressed by the two articles in this issue from different, yet complementary, perspectives by presenting experiences from two projects supported by national funding programmes [5].

Biddle et al. describe an approach based on a targeted, group-specific sampling and recruitment which enables health monitoring among refugees living in collective accommodation by integrating them into health surveys. Jahn et al. describe an innovative approach to using routine medical data in reception facilities, which is based on the principle of distributed computing. Both approaches create new information resources that enable the integration of the target group in terms of the visibility of relevant health aspects in settings that have not yet been systematically considered. However, sustained use of this information in health reporting will require a structural consolidation of these approaches at national, federal state and municipal levels.
